# Efficacy of Bacopa monnieri (Linn.) on Cognitive Function and Alterations in Blood Metabolites in Patients With Amnestic Mild Cognitive Impairment and Early Alzheimer Disease: Protocol for an Exploratory Double-Blind, Randomized, Placebo-Controlled Trial

**DOI:** 10.2196/82891

**Published:** 2026-02-25

**Authors:** Abhilasha Dwivedi, Anjali Anjali, Hina Narzari, Yashwant Kumar, Hanuman Prasad Sharma, Aditi Dubey, Nilima Nilima, Roopa Rajan, Mamta Bhushan Singh, Venugopalan Y Vishnu, Rohit Bhatia, Gautam Sharma, Anu Gupta

**Affiliations:** 1 Department of Neurology, Faculty of Neurology, All India Institute of Medical Sciences, 7th Floor, Room no. 708, New Delhi, 110029, India, 91 8929466868; 2 Department of Biostatistics, All India Institute of Medical Sciences, New Delhi, India; 3 Noncommunicable Diseases, Translational Health Science and Technology Institute, Faridabad, India; 4 Bioanalytics Facility, Centralized Core Research Facility, All India Institute of Medical Sciences, New Delhi, India; 5 Department of Cardiology, Incharge Center for Integrative Medicine and Research, Faculty of Cardiology, All India Institute of Medical Sciences, Delhi, India

**Keywords:** *Bacopa monnieri* (Linn.), mild cognitive impairment, MCI, amnestic, Alzheimer disease, AD, metabolomics

## Abstract

**Background:**

Amnestic mild cognitive impairment (aMCI) represents a transitional stage between normal aging and Alzheimer disease (AD), where early intervention is critical for preserving cognition and delaying or preventing progression to dementia. Due to the limited availability of curative pharmacological treatments, there is growing interest in traditional and indigenous medicinal interventions, such as *Bacopa monnieri* (Linn.) or Brahmi, a widely used Ayurvedic nootropic herb. *Bacopa* consumption is known to enhance cognitive performance in healthy individuals and is associated with alterations in pathways related to branched-chain and aromatic amino acid biosynthesis. These pathways have been implicated in MCI due to AD pathophysiology. Hence, the efficacy of *Bacopa* in aMCI and the mediating metabolic changes need to be systematically evaluated through clinical trials.

**Objective:**

The aim of this study is to assess the effects of *B. monnieri* on cognitive function and plasma metabolites in individuals with aMCI (early or prodromal AD).

**Methods:**

This study will employ a double-blind, randomized, placebo-controlled trial design, with 76 participants (38 per group) of aMCI diagnosed using the clinical National Institute on Aging–Alzheimer’s Association 2011 criteria in a tertiary care setting in India. Participants will receive either *B. monnieri* (Linn.) (300 mg standardized plant extract) or a matched placebo daily for 12 weeks. Comprehensive cognitive assessments (0, 12, and 24 wk) and untargeted plasma metabolomic profiling (0 and 12 wk) will be conducted to evaluate both cognitive changes and alterations in plasma metabolites. MetaboAnalyst 6.0 (with integrated features like Kyoto Encyclopedia of Genes and Genomes and the Human Metabolome Database) will be used for a comprehensive statistical and functional analysis pertaining to the metabolomics. The primary outcome will be a change in the composite *z* score of memory between the 2 groups at 12 weeks. The secondary outcomes will include alterations in metabolites and pathways, and adverse events at 12 weeks, and cognitive performance at 12 and 24 weeks.

**Results:**

The study was funded in October 2023, and the first participant was enrolled in April 2024. As of November 2025, a total of 60 participants have been recruited, with a mean (SD) age of 62.2 (8.1) years. The cohort predominantly comprises men (53/60, 88.3%), individuals with at least a high school education (47/60, 78.3%), and participants at elevated risk for cognitive decline. Specifically, 39 of 60 participants (65%) have diabetes, 29 (48.3%) have hypertension, and 13 (21%) report a positive family history of dementia. Data collection will conclude in June 2026, after which data analysis will begin and be completed by September 2026, with primary findings targeted for publication in spring 2027.

**Conclusions:**

This protocol investigates the efficacy of *B. monnieri* (Linn.) in improving cognitive function and altering the blood metabolites in patients with aMCI. If effective, this intervention could provide an accessible and cost-effective approach to manage early AD in resource-limited settings.

## Introduction

### Background

Mild cognitive impairment (MCI), an intermediate clinical state between cognition of normal aging and mild dementia, is characterized by subjective cognitive complaints and objective impairment in one or more cognitive domains, such as memory, executive function, visuospatial skills, and language, while independence in instrumental activities of daily living is preserved [1]. The estimated prevalence of MCI in India is 17.6%, while the prevalence of major neurocognitive disorder such as Alzheimer disease (AD) is 7.2% [2]. Worldwide, MCI affects approximately 15% to 20% of older adults, while AD affects about 5% to 7% of the global population aged 60 years and above [3]. Considered the prodromal stage of dementia, MCI is a critical window period for early therapeutic intervention.

MCI has 2 clinical subtypes: amnestic mild cognitive impairment (aMCI) and nonamnestic MCI. aMCI is characterized by significant memory impairment, while in nonamnestic MCI, individuals face challenges with language, executive functions, or visuospatial skills [4]. aMCI subtypes are often linked to AD and pose a significantly higher risk of progression toward dementia [5]. Recently, antiamyloid therapies have received approval for MCI due to AD, but these drugs are costly, require sophisticated imaging or fluid tests before administration, and will take time to align to routine clinical practice [6]. The other US Food and Drug Administration approved medications for AD, such as donepezil (a cholinesterase inhibitor) and memantine (an N-methyl-D-aspartate receptor antagonist), are not approved for use in MCI. These medications also have numerous adverse effects, such as gastrointestinal effects (nausea, vomiting, diarrhea, loss of appetite), vagotonic (bradycardia, heart block), headache, confusion, and bladder incontinence [7,8]. In this scenario, traditional interventions for tackling cognitive decline are gaining focus.

The Indian system of medicine or Ayurveda (Sushruta Samhita, Charak Samhita, and Atharva Veda) describes plants that have a specific action on the intellect and memory as “Medhya Rasayana.” The word “Medhya” stands for “intellect or retention,” while “Rasayana” means “procedure or preparation” [9]. *Bacopa monnieri* (known as “Brahmi” in India), a herbal nutraceutical native to South and Southeast Asia, is recognized for this role and has been traditionally used for memory loss, anxiety, epilepsy, and insomnia [10]. Its leaves contain important natural compounds such as triterpenoid saponins (bacoside A and B), alkaloids, flavonoids, and phytosterols, with bacosides being essential for its effects on the brain, which are known to be pleiotropic. Numerous experimental studies have shown that *Bacopa* protects cholinergic neurons [11,12], upregulates expression of N-methyl-D-aspartate receptors (which mediate structural changes that occur in neurons during learning and memory formation) in the prefrontal cortex and hippocampus [13], reduces stress-induced hippocampal damage [14], enhances cerebral blood flow [15], increases expression of neuroplasticity markers like brain-derived neurotrophic factor and Arc protein expression [16], and has an antioxidant and free radical scavenging action [17-19]. It also has an anti-amyloidogenic potential, reducing formation of amyloid fibrils and offering protection against neuronal cell death induced by amyloid-β protein [20-22].

Looking at the clinical translation of the above experimental studies, Kongkeaw et al [23] did a meta-analysis of 9 randomized controlled trials (RCTs) where standardized extracts of *B. monnieri* were administered to 437 eligible participants (heterogeneous study population in terms of age range; most were healthy volunteers, except for 79 participants who had memory complaints) for at least 12 weeks. The authors reported a significant improvement in cognitive processing speed after 12 weeks of *B. monnieri* extract. However, the effect of *Bacopa* on the memory domain was inconclusive because of high heterogeneity. Subsequent studies using *Bacopa* in healthy older adults have reported improvement in working memory, a decrease in N100 (assessing selective attention) and P300 latencies (assessing cognitive processing) of event-related potentials, and suppression of plasma acetylcholinesterase activity [24].

To evaluate the role of *Bacopa* in people with cognitive impairment, Basheer et al [25] did a meta-analysis on the effectiveness of *B. monnieri* in mild, moderate, or severe dementia due to AD or MCI-AD. The authors found no difference between *B. monnieri* and the placebo or donepezil. The analysis included 5 eligible studies; 2 compared *Bacopa* with donepezil, and the rest compared Bacopa with placebo. There was heterogeneity in dosage, duration, formulation, follow-up, and outcomes, and the overall quality of evidence was very low due to high risk of bias, small sample size, and wide CIs. However, no major safety issues were reported in the included trials.

Recently, Minale et al [26] used liquid chromatography-mass spectrometry (LC-MS) in healthy individuals (age range, 55‐80 y) who had consumed *Bacopa* extract for 12 weeks and showed changes in metabolites (aminoacyl-transfer RNA biosynthesis, aromatic and branched-chain amino acid biosynthetic pathways) in plasma, urine, and feces. Prior research findings on blood and cerebrospinal fluid metabolites in AD and MCI have suggested possible alterations in metabolomic pathways associated with cellular energy and biosynthesis, oxidative stress, amino acid utilization, lipid homeostasis, mitochondrial oxidative metabolism, and neurotransmitters [27-31]. Yilmaz et al [32] combined proton nuclear magnetic resonance spectroscopy, plasma LC-MS, and machine learning statistical approaches in a community-based sample cohort acquired from different sites across the United States and reported significant perturbations in amino sugar, alanine, glucose-alanine cycle (energy supply to muscles and nitrogen disposal), cysteine and aspartate metabolism, and urea cycle in patients with MCI compared with healthy controls. Our recent work using untargeted metabolomics in Indian patients with AD (vs cognitively normal controls) showed disruptions in branched-chain amino acid metabolism, nicotinamide metabolism, the tricarboxylic acid cycle, and neurotransmitter pathways [33]. Although the study by Minale et al [26] in healthy subjects suggests that *Bacopa* may affect pathways implicated in MCI and AD, it needs further validation.

### Need for the Study

aMCI has a high risk of progression to Alzheimer dementia. Currently, there are few approved treatments for aMCI. Antiamyloid agents are the only approved pharmacological options and need sophisticated tests for accurate diagnosis of amyloid pathology in the brain. These drugs are costly, need monitoring for serious adverse effects (SAEs), and are not yet available in low- and middle-income countries. *B. monnieri*, a herb that has been used as a memory enhancer for centuries in India, is a culturally aligned intervention, cheap, and without SAEs. Most studies in the literature on the effectiveness of *Bacopa* on cognitive functions have focused on healthy individuals. Reports in cognitively impaired older adults are few and provide low-certainty evidence. Furthermore, few studies have explored the underlying biological mechanisms of *Bacopa* using the modern “omics” approaches. This study aims to fill these critical gaps by assessing the efficacy of *B. monnieri* (Linn.) in improving memory and other cognitive functions in individuals with aMCI (early or prodromal AD) through an exploratory double-blind randomized controlled trial design. It will also examine related changes in blood metabolites through untargeted metabolomics. We will use a standard *Bacopa* extract containing 50% bacosides, a matching placebo, and culturally valid cognitive tests. The results may contribute to therapeutic strategies for this entity and promote evidence-based traditional medicine within mainstream health care.

### Objectives

The primary objective of the study is to evaluate the effect of *B. monnieri* (Linn.) on memory as compared with placebo among individuals with aMCI after 12 weeks of intervention. The secondary objectives are to identify alterations in metabolites, pathways, and networks after administration of *B. monnieri* (Linn.) and to evaluate the effect on other cognitive domains (processing speed, attention, and executive functions) at 12 and 24 weeks. The study tests the hypothesis that *B. monnieri* (Linn.) (300 mg standardized whole-plant extract) is more effective than placebo in improving the memory in patients with aMCI over 12 weeks.

## Methods

### Study Design

This study will follow a double-blind, randomized, placebo-controlled trial design with 3 phases of assessment: baseline evaluation (week 0), postintervention assessment at 12 weeks, and follow-up assessment at 24 weeks.

### Study Settings

The study will be conducted at the outpatient services of the Department of Neurology, All India Institute of Medical Sciences (AIIMS), New Delhi, a tertiary academic and research institute in India. Participants presenting with memory complaints will be screened and recruited by qualified neurologists and professional clinical psychologists based on predefined inclusion and exclusion criteria. Eligible participants will be enrolled in the study and randomly assigned to 1 of 2 groups using a stratified block randomization method (stratified by age: 45‐60 and 61‐75 y) with a fixed block size of four and a 1:1 allocation ratio. Participants will receive either *B. monnieri* (300 mg standardized whole-plant extract) or a matched placebo, taken once daily with water after breakfast for 12 weeks. All clinical assessments, blood sample collections, and follow-up visits will be done at the designated study location.

### Study Population (Inclusion Criteria)

The trial will recruit participants aged 45 to 75 years who have a clinical diagnosis of aMCI or early AD, diagnosed using the clinical criteria given by the National Institute of Ageing-Alzheimer’s Association workgroup (2011) [34]. Participants should report a decline in memory compared with previous ability, have impaired scores (≤15th percentile on the published norms as abnormal) on tests of verbal or visuospatial memory on the detailed neuropsychological battery, and preserved functional independence [35]. They should be willing to participate in the study, give written informed consent, be able to comply with the study requirements, read and comprehend either English or Hindi for cognitive test assessments, and should have at least 5 years of formal school education (participants with low education may not perform as well on neuropsychological testing) [36].

### Exclusion Criteria

Participants with advanced malignancies; significant systemic diseases such as chronic kidney disease, severe hepatic, pulmonary, cardiac disease; other neurologic diseases or drugs that are known to affect cognition (like structural brain lesions, stroke, epilepsy, traumatic brain injury, Parkinson disease, Parkinson-plus syndromes, normal pressure hydrocephalus, autoimmune/paraneoplastic encephalitis); history of a major surgical procedure in the last 6 months; uncontrolled hypothyroidism (thyroid-stimulating hormone >10 µU/ml at time of enrollment) will be excluded. Participants with a clinically diagnosed major psychiatric disorder (eg, psychosis, major depression, bipolar disorder), those who use psychoactive medications that could affect their ability to reliably perform neurocognitive testing, have visual or auditory impairment that would preclude the participant from participating in or cooperating with the protocol, have a history of intake of *B. monnieri* or any other investigational drug or cholinesterase inhibitors within 1 month prior to screening visit, or with alcohol abuse as defined by the National Institute on Alcohol Abuse and Alcoholism will also be excluded. The National Institute on Alcohol Abuse and Alcoholism defines heavy drinking as follows: for men, consuming more than 4 drinks on any day or more than 14 drinks per week; for women, consuming more than 3 drinks on any day or more than 7 drinks per week [37].

### Recruitment and Baseline Assessment

**Figure 1.**
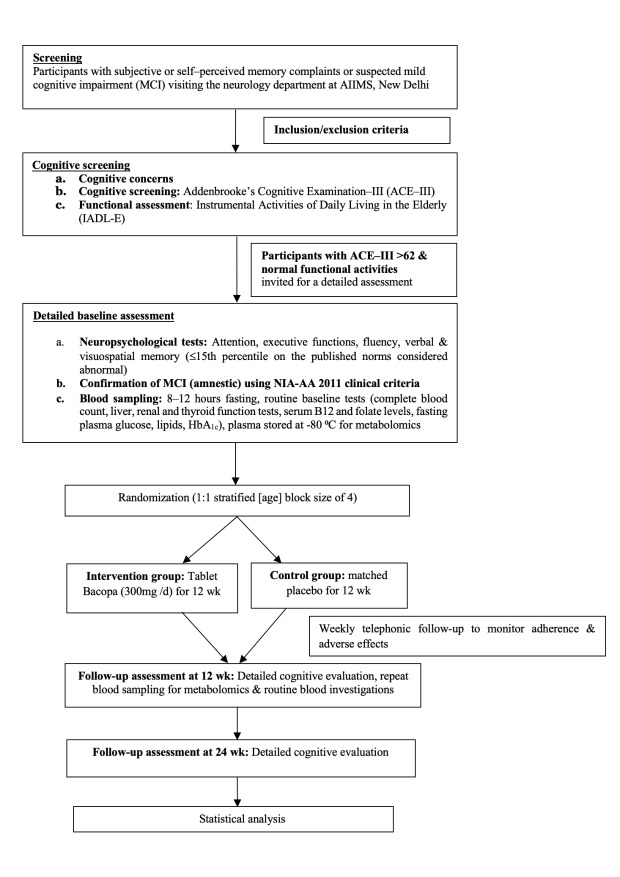
Study flow. Inclusion criteria included: aged 45-75 years, with a clinical diagnosis of amnestic mild cognitive impairment (NIA-AA, 2011 clinical criteria), willing to participate and give written informed consent, able to read and comprehend either English or Hindi, and with at least 5 years of formal school education. Exclusion criteria included: participants with advanced malignancy, significant systemic diseases like chronic kidney disease, severe hepatic, pulmonary, cardiac disease, other neurologic diseases or drugs known to affect cognition, major surgical procedure in the last 6 months; thyroid-stimulating hormone >10 µU/ml at time of enrollment; major psychiatric disorder (eg, psychosis, major depression, bipolar disorder); use of psychoactive medications; visual or auditory impairment; history of intake of *Bacopa monnieri* or any other investigational drug or cholinesterase inhibitors within 1 month prior to screening visit; or presence of alcohol abuse (National Institute on Alcohol Abuse and Alcoholism guidelines). AIIMS: All India Institute of Medical Sciences; NIAAA: National Institute on Alcohol Abuse and Alcoholism; NIA-AA: National institute on Ageing-Alzheimer’s Association.

Eligible participants will be identified by neurologists and clinical psychologists. Participants meeting the inclusion and exclusion criteria will undergo cognitive screening using the Indian adaptation of the Addenbrooke’s Cognitive Examination III (ACE-III; Hindi version) [38] and functional screening Instrumental Activities of Daily Living in the Elderly scale[39]. Participants with an ACE-III score greater than 62 [40] and a cognitive disability index ≤16 will be invited for a detailed cognitive assessment. This excludes mild dementias and ensures that all the recruited participants are at the MCI stage. A structured questionnaire will be used to gather information on sociodemographic characteristics, medical history, concomitant medications, vital signs, lifestyle factors, and risk factors (Table S1 in Multimedia Appendix 1). Cognitive assessments will be conducted by trained clinical psychologists using the Indian Council of Medical Research-Neurocognitive Tool Box [35], a culturally validated tool for diagnosis of MCI in India (Table 1). The study flow is presented in Figure 1.

**Table 1.** Details of cognitive assessment.

Cognitive domain	Test
Attention and executive functioning [35]	
Visuospatial attention, concentration, and set shifting	Trail making test (black and white)
Verbal fluency	Category fluency (animal, food, vegetable); phonemic fluency (Ka, Ma, Pa)
Episodic memory	
Verbal memory	Verbal learning test (total learning, learning over trials, delayed recall, and delayed recognition)
Visuospatial memory	Test des Neuf Images du—93 (TNI-93; immediate recall, free recall, cued recall and spatial recall)
Global cognition	
Screening of all domains	Addenbrooke’s Cognitive Examination III
Functional activities	
Instrumental activities of daily living	Instrumental Activities of Daily Living–Elderly

### Blood Sample Collection and Storage

Venous blood samples will be collected by certified laboratory technicians. Participants will be instructed to maintain an 8 to 12 hours fast and to abstain from alcohol for at least 72 hours before sample collection. Blood will be drawn aseptically into the following tubes: 2 plain tubes, 3 ethylenediaminetetraacetic acid tubes, and 1 gray-top (fluoride) tube. Samples showing signs of hemolysis will be excluded from further analysis. The collected samples will be used for routine biochemical investigations, including complete blood count, glycated hemoglobin (HbA_1c_), liver function tests, kidney function tests, lipid profile, thyroid profile, fasting blood glucose, vitamin B12, and folate levels in serum. For metabolomic analysis, 1 plain and 1 ethylenediaminetetraacetic acid tube will be used, and the samples will be transported via cold chain. All samples will be centrifuged at 2500 rpm for 15 minutes at room temperature to separate serum and plasma within 1 hour of drawing the sample. The separated components will be aliquoted into cryovials and stored immediately at –80 °C until further analysis.

### Randomization

Participants will be randomly assigned to the intervention or control group using a computer-generated list. As age is the most significant risk factor for cognitive decline, and our study has a relatively small sample size, we will do a stratified randomization by age (45‐60 and 61‐75 y) to avoid significant baseline differences pertaining to age in the 2 groups. The sequence will be created using Stata 17 by an independent statistician. Unique IDs starting with “BRY2001-BRY2038” for younger and “BRE3001-BRE3038” for older participants will be assigned prior to allocation to ensure a systematic and unbiased process. To maintain a 1:1 allocation ratio and minimize selection bias, a block size of 4 will be applied within each age stratum. This approach ensures that participants in both age groups are evenly distributed between the intervention and placebo arms. To prevent selection bias, the randomization list will be kept strictly confidential and accessible only to the independent statistician, who will not be involved in recruitment or data collection.

### Allocation Concealment

The statistician generating the randomization list will prelabel the medicine bottles (intervention and placebo) with sequential IDs based on the randomization list. These prelabeled bottles will be used during participant enrollment, ensuring that the recruitment team remains unaware of group assignments at all times. This process ensures that the allocation remains concealed before and during assignment, maintaining the integrity of the randomization process.

### Blinding

This study will adopt a double-blind design, in which participants, caregivers, and investigators including those administering the intervention, conducting assessments, and handling data are blinded to group allocation throughout the study. This blinding helps reduce both performance and detection bias, supporting the reliability and objectivity of the trial results.

### Intervention Arm

Participants will receive either the *B. monnieri* (Linn.) tablets (300 mg) or a matched placebo, identical in appearance, weight, shape, size, color, and packaging. *B. monnieri* (Linn.) will be administered as a standardized whole-plant extract at a dose of 300 mg once daily, taken orally in the morning after meals with water. At baseline, each participant will be provided with a bottle containing 90 tablets, along with 6 additional tablets to account for potential missed doses. The *B. monnieri* (Linn.) formulation (Dabur India Limited) contains 50% bacosides, and the placebo will be devoid of active bacosides while maintaining physical similarity. The intervention period will last for 12 weeks. Concomitant medications for comorbidities will be allowed, and any change in the prescriptions will be documented.

### Control Arm

The placebo tablets will be carefully matched with the investigational tablets in terms of color, taste, texture, shape, and size, and will be supplied in similar bottles. Placebo tablets will be prepared using starch and the same excipients that are known to be biologically inactive. Each participant will be given dispensed bottles (containing 90 tablets) at the time of the baseline visit.

### Methodology for Metabolomics

This study will use the LC-MS approach for untargeted metabolite analysis.

#### Sample Preparation for Protein Extraction

Prior to LC-MS/MS analysis, plasma samples will be deproteinized using a protein precipitation technique. Nonaqueous solvents such as chilled methanol will be used to remove proteins. Plasma samples will be thawed on ice and be vortexed using a vortex mixer to ensure uniformity. For each sample, 100 μl of plasma will be transferred into a new 2-ml microcentrifuge tube, followed by the addition of 400 μl of chilled methanol. The mixture will be vortexed for 1 minute and then centrifuged at 10,000 rpm for 10 minutes at 4 °C. After centrifugation, the clear supernatant will be carefully transferred into new 2 ml microcentrifuge tubes in a fixed volume. These samples will then be subjected to vacuum drying using a CentriVap Cold Trap vacuum concentrator (Labconco, Kansas City, MO, USA). Finally, each sample will be resuspended in 25 μl of a methanol-water mixture (3:17, methanol:water), vortexed briefly, and centrifuged at 11,000 rpm for 10 min at 4 °C and injected into LC-MS/MS.

#### LC-MS/MS Methodology

The Orbitrap Fusion mass spectrometer (Thermo Scientific) coupled with a heated electrospray ion source will be used for data acquisition. Data acquisition methods have been followed as per published protocols [41,42] with minor modifications. Briefly, for MS1 mode, mass resolution will be kept at 120,000, and for MS2 acquisition, mass resolution will be 30,000. The mass range of data acquisition will be 60 to 900 Da. Extracted metabolites will be separated on UPLC Ultimate 3000. Data will be acquired on reverse-phase and hydrophilic interaction liquid chromatography columns in both positive and negative ionization modes. HSS T3 will serve as a reverse phase column, while XBridge BEH Amide (Waters Corporation) will be used as a hydrophilic interaction liquid chromatography column. For polar compound separation, solvent A will have 20 mM ammonium acetate in the water (pH 9), and mobile phase B will have 100% acetonitrile. The elution gradient will start from 85% B to 10% B over 14 min with a flow rate of 0.35 ml/min. For reverse phase, solvent A will be water, and B will be methanol with 0.1% formic acid added in both solvents. The elution gradient will start with 1% B to 95% B over 10 min with a flow rate of 0.3 ml/min. The sample injection volume will be 5 µl. Pooled quality control samples will run after every five samples to monitor signal variation and drift in mass error.

### Data Processing

All LC-MS–acquired data will be processed using the Progenesis QI for Metabolomics (Water Corporation) software using the default settings. The untargeted workflow of Progenesis QI facilitates retention time alignment, feature detection, deconvolution, and prediction of elemental composition. The Metascope plug-in of Progenesis QI will be used for the in-house library, incorporating accurate mass, fragmentation patterns, and retention times for database searches. The study will use an online spectral library for further confirmation of identification. The retention time match cut-off will be set at 0.5 minutes, with a spectral similarity exceeding 30% for fragmentation matches in Progenesis QI. Peaks with a coefficient of variation below 30% in pooled quality control samples will be chosen for subsequent data analysis. Furthermore, manual verification of each identified feature will be conducted to select the appropriate peaks.

### Follow-Up

There are 3 in-person primary data collection points in the study: “baseline evaluation (week 0),” “postintervention assessment at 12 weeks,” and “second follow-up assessment at 24 weeks.” Between the in-person visits, participants will receive weekly telephonic follow-up calls to promote participant retention, monitor medication adherence, and collect information on any SAEs. In the case of any SAEs, participants will be instructed to discontinue the study medication immediately and to contact the study team for appropriate medical evaluation. In such scenarios, unblinding will be done by the statistician who has access to the randomization and conveyed to the treating medical team. Following completion of the 12-week intervention period, participants will return to the hospital for a comprehensive follow-up visit, which will include assessment of primary and secondary outcome measures; and blood sample collection for postintervention metabolomic analysis, and repeat laboratory investigations, including hematological, hepatic, and renal function tests. Participants will be allowed to withdraw from the study if they wish to do so, and the reason for withdrawal will be clearly documented. They will be encouraged to return the used bottles at the end of 12 weeks or if they withdraw from the study at any time point, for pill count to confirm adherence. The second follow-up assessment will take place at 24 weeks and will include detailed cognitive evaluation. Blood sampling will not be done at this time point. Participants will continue to receive posttrial standard of care from their treating physicians.

### Outcomes

The primary outcome will be change in the composite *z* score of memory between the 2 groups at 12 weeks. The memory domain will include both verbal (Verbal Learning Test: learning over trials, delayed recall, delayed recognition) and visuospatial memory (Test des Neuf Images du 93-Spatial Recall). The secondary outcomes will include alterations in metabolites, pathways, and molecular networks, and frequency of SAEs after 12 weeks of treatment with *B. monnieri*; change in *z* scores for other cognitive domains (attention, executive functioning, and information processing) at 12 and 24 weeks (including *z* score for the memory domain at 24 wk); proportion of participants in both groups who demonstrate clinically meaningful cognitive improvement, defined as either ≥5 point improvement on the ACE-III [43], or ≥0.5 SD improvement in the composite *z* score of memory domain in the *Bacopa* versus placebo group at 12 and 24 weeks. Adherence will be monitored through self-reported medication logbooks and weekly telephonic follow-ups.

### Ethical Considerations

This study has received ethical approval from the Institute Ethics Committee of the All-India Institute of Medical Sciences (AIIMS), New Delhi, India (reference IEC–660/03.09.2021, RP–35/2021). Written informed consent will be obtained from all participants before their enrollment in the study by the clinical psychologist under the supervision of the principal investigator. Participants will be told about the study’s goals, methods, risks, and benefits, as well as their right to withdraw and the steps taken to keep their data confidential. Data will be entered into REDCap (Research Electronic Data Capture), which is a secure web application for managing databases. Any data sharing will involve anonymization of personally identifiable information. All procedures will adhere to the ethical standards established by the institutional review board and the principles outlined in the Declaration of Helsinki. This is an academic, investigator-initiated, nonpharma-funded clinical trial; hence, there is no provision of compensation in the study protocol. However, any adverse event related to the research will be treated free of cost in the hospital.

### Statistical Methods

#### Sample Size Estimation

There are no previous studies investigating the effects of *B. monnieri* versus placebo in patients with aMCI, looking at changes in composite memory *z* scores as the primary outcome. By considering a clinically relevant effect size as a SD change of 0.5, a 2-sided α at 5%, and 80% power, a sample size of 128 (64 in each group) was calculated. With a 20% dropout rate, the final sample size reached 160 (80 in each group), which did not seem feasible for the selected patient population in the specified duration. Therefore, a sample size of 60 (30 in each group) is determined for this exploratory study based on feasibility. The sample size was further adjusted for a dropout rate of 20% and 76 participants (38 in each group) will be recruited for this study.

#### Planned Analyses

As mentioned previously, data will be entered into REDCap, a secure web-based application for managing databases. A data monitoring committee is not needed for this trial, as it is a short-duration study and the known risks of the intervention drug are minimal. No interim analysis is planned. Data will be analyzed using Stata version 17.0 (StataCorp LLC, College Station, TX, USA) and presented data as number (%), mean (SD), or median (IQR), as appropriate. Pearson chi-square test or Fisher exact test will be used (as appropriate) for qualitative variables. For quantitative variables, normality will be assessed using the Shapiro-Wilk test. The Student *t* test for independent samples will be used to compare differences in means for normally distributed quantitative variables. The Mann-Whitney *U* test will be used for quantitative variables with a distribution other than normal. All tests will be 2-tailed, and a *P* value <.05 will be considered statistically significant. Raw scores of each cognitive test will be converted to a *z* score (*z* score=[individual participant raw score – group mean]/group SD) for each participant. The group mean and SD will be derived by pooling the baseline scores of all participants. For all the tests, a higher *z* score will indicate better performance, except for the trail making tests A and B. The signs of these two tests will be inverted for a uniform interpretation. Both intention-to-treat and per protocol analysis will be done. Generalized linear mixed models with a between-subject factor of group (treatment vs placebo) and within-subject factor of time (baseline, mid-point, end point) will be conducted to assess differences in continuous primary and secondary outcome measures. Planned simple contrasts will be conducted to assess all time points (baseline vs. 12 wk, 12 wk vs. 24 wk, and baseline vs 24 wk). To handle any missing data, analysis of last observation carried forward will be undertaken within the intention-to-treat analysis model. Comprehensive statistical and functional analysis pertaining to the metabolomics will be performed using MetaboAnalyst version 6.0. The investigation based on observed peak values will encompass principal component analysis, partial least squares discriminant analysis, ANOVA, and molecular pathway identification. Heat maps will be developed with samples arranged in columns and features in rows, presented in a gradient from cold to hot. Pathway analysis will be conducted utilizing the integrated features of MetaboAnalyst 6.0, which includes the Kyoto Encyclopedia of Genes and Genomes and the Human Metabolome Database.

### Reporting Guidelines

The SPIRIT 2025 reporting guidelines have been used while reporting this study protocol. The checklist is provided in Checklist 1. Changes made to protocol after submission to CTRI are summarized in Table S2 in Multimedia Appendix 1.

### Dissemination of Results

The results will be presented at scientific meetings and will be published in scientific journals. The results will also be conveyed to the public in simple language during patient awareness programs after study completion.

## Results

### Study Status and Timeline

The study was funded in October 2023, and the first participant was enrolled in April 2024. Data collection will conclude in June 2026, after which data analysis will begin and be completed by September 2026, with primary findings targeted for publication in spring 2027.

### Baseline Characteristics

As of November 2025, a total of 60 participants had been recruited, with a mean age of 62.2 (SD 8.1) years. Most participants were men (53/60, 88.3%), and 47/60 (78.3%) had completed at least high school (Table 2). Among the risk factors, 39/60 (65%) had diabetes, 33/59 (55.9%) had dyslipidemia, 29/60 (48.3%) had hypertension, 29/60 (48.3%) were overweight or obese, 15/60 (25%) had coronary artery disease, and 13/60 (21%) reported a positive family history of dementia (Table 3). The biochemical profile and the cognitive raw scores are provided in Tables S3 and S4 in Multimedia Appendix 1, respectively.

**Table 2.** Baseline sociodemographic characteristics of the participants.

Characteristics	Whole cohort (N=60)
Age, y, mean (SD)	62.2 (8.1)
Men, n (%)	53 (88.3)
Married, n (%)	57 (95)
Education (high school and above), n (%)	47 (78.3)
Occupation, n (%)	
Unemployed	3 (5)
Semiskilled and skilled workers	27 (45)
Semiprofessional to professional	16 (26.7)
Arithmetic skill job	14 (23.3)
Monthly family income (INR^a^), mean (SD)	37,462.5 (18,584.7)
Socioeconomic status, n (%)	
Upper class	37 (61.7)
Middle class to upper-middle class	20 (33.3)
Lower class to lower-middle class	3 (5)

a 1 INR equals US $0.011.

**Table 3.** Baseline risk factor profile of the participants.

Characteristics	Whole cohort (N=60)
Height (cm), mean (SD)	163.7 (6.9)
Weight (kg), mean (SD)	68.4 (10.2)
Body mass index (kg/m²), mean (SD)	25.6 (3.8)
Average SBP^a^ (mm Hg), mean (SD)^b^	126.7 (16.5)
Average DBP^c^ (mm Hg), mean (SD)^b^	75 (10.5)
Pulse rate (bpm), mean (SD)	92.5 (11.8)
Duration of forgetfulness (months), mean (SD)	40.8 (23.6)
Hypertension, n (%)	29 (48.3)
Duration of hypertension (months), median (IQR)	72 (3-138)
Diabetes, n (%)	39 (65)
Duration of diabetes (months), median (IQR)	96 (36-186)
CAD^d^, n (%)	15 (25)
Duration of CAD (months), median (IQR)	120 (90-162)
Dyslipidemia, n/N (%)	33/59 (55.9)
Overweight, n (%)	20 (33.3)
Obese, n (%)	9 (15)
Hypothyroidism, n (%)	3 (5)
Hyperthyroidism, n (%)	5 (8.3)
Alcohol use, n (%)^e^	3 (5)
History of smoking, n (%)	0 (0)
Family history of dementia, n (%)	13 (21)

a SBP: systolic blood pressure.

b Average of 3 readings taken 2 minutes apart.

c DBP: diastolic blood pressure.

d CAD: coronary artery disease.

e Not fulfilling the National Institute on Alcohol Abuse and Alcoholism criteria of alcohol abuse.

### Deviations From Original Registered Protocol

We made some amendments to the inclusion and exclusion criteria, allocation concealment procedure, and cognitive test battery before the commencement of participant recruitment (December 2023). These revisions were minor and intended to improve implementation, feasibility, and the precision of cognitive assessments. They did not affect the overall study design, sample size, randomization strategy, or predefined clinical outcomes. These deviations are summarized in Table S2 in Multimedia Appendix 1. We have added “at least 5 years of formal school education” to the inclusion criteria, and “participants who have undergone a major surgical procedure in the last 6 months, or thyroid-stimulating hormone >10 µU/ml at time of enrollment” to the exclusion criteria. We also elaborated on “other neurologic diseases or drugs known to affect cognition” (eg, structural brain lesions, stroke, epilepsy, traumatic brain injury, Parkinson disease, Parkinson-plus syndromes, normal pressure hydrocephalus, autoimmune/paraneoplastic encephalitis) in the exclusion criteria. Prelabeled medicine bottles (intervention and placebo) with sequential IDs based on the randomization list are being used for allocation concealment instead of “sequentially numbered opaque sealed envelopes.” Two changes were made to the cognitive battery: ACE-III (clinically significant improvement will be defined as ≥5-point improvement in score) is being used for cognitive screening and Test des Neuf Images du 93 for visuospatial memory in place of Montreal Cognitive Assessment scale and Modified Taylor Complex Figure Test, respectively.

## Discussion

### Principal Findings

The current clinical trial seeks to evaluate the potential of *B. monnieri* (Linn.) as a therapeutic intervention for middle-aged and older adults (aged 45‐75 y) diagnosed with aMCI. Previous literature on the efficacy of *B. monnieri* in enhancing cognitive functions has shown variable results. Most clinical studies that support its efficacy for improving memory and other cognitive functions have been done in healthy adults [23,24]. There is limited data on its efficacy in aMCI. A Cochrane review on the clinical efficacy and safety of *B. monnieri* in persons with mild, moderate, or severe dementia due to AD or with MCI-AD by Basheer et al [25] found a high risk of bias in the included trials, and imprecise results due to small sample sizes (most studies had 30‐40 participants in total) and wide CIs. There was heterogeneity in the formulation (some studies have used *Bacopa* in combination with herbal extracts, others have used *Bacopa* extract only), comparator (donepezil or placebo), treatment duration, follow-up, and outcomes. We have summarized the randomized controlled trials in which *B. monnieri* has been used for healthy older adults, or MCI, or AD dementia in Table S5 in Multimedia Appendix 1.

Very few studies have reported the efficacy and safety of *Bacopa* in different dosages and formulations. Most studies have compared a dried extract composed of 300 to 320 mg of *Bacopa,* either once a day or in two divided dosages, against placebo and reported efficacy with no major side effects [24,26,44,45]. Others have used doses ranging from 250 to 450 mg and 600 mg [11,46-48]. Studies have not demonstrated any incremental benefit with a higher dose [24].

From a safety perspective, Pravina et al [49], in a phase I study, evaluated the short-term safety and tolerability of *B. monnieri* in healthy adult volunteers (aged 22‐42 y) through a randomized, open-label, dose-escalation design. The standardized extract of *B. monnieri* was given to each participant in a dose of 300 mg per day for 15 days, and then 450 mg per day for another 15 days. Pre- and post-treatment examination of clinical, hematological, biochemical, and electrocardiographic parameters of the treated volunteers did not indicate any untoward effects. There were mild adverse events related to the gastrointestinal system in the trial (300 mg dosage: epigastric burning sensation in one volunteer, nausea in one volunteer; 450 mg dosage: fullness and bloating sense of abdomen in one volunteer) which resolved spontaneously without any need to discontinue treatment. There were no dropouts in the study period. At the dose administered for the given duration of the trial period, *B. monnieri* extract was found to be safe in healthy adults. Stough et al [50] have also reported a higher percentage of participants with nausea (18% vs 4%), dry mouth (23% vs 16%) and fatigue (14% vs 4%) in the *B. monnieri* (300 mg for 12 wk) group in comparison to placebo (double-blind placebo-controlled study in 46 healthy volunteers aged between 18 and 60 y). Other studies have reported rash, constipation, drowsiness, and aftertaste following ingestion of *B. monnieri* formulation [25]. Prabhakar et al [51] in their study comparing *Bacopa* to donepezil reported 3 deaths due to myocardial infarction (2 in the donepezil arm and 1 in the *B. monnieri* arm).

Given the complexity of AD pathophysiology, omics-based investigations of molecular and cellular pathway disruptions are gaining momentum, as they can guide biomarker and therapeutic development. From a mechanistic perspective, *B. monnieri* has been postulated to have antiapoptotic, antioxidant actions, repairs damaged neurons, stimulates kinase activity, restores synaptic function, and improves neurotransmission. A systematic review of 22 studies reported that *B. monnieri* reduces the levels of proinflammatory markers, oxidative stress, and also reduces nuclear factor-κB phosphorylation [52]. However, there is a lack of adequate clinical research on the molecular targets and pathways of *Bacopa* in modulating neuroinflammation and neurotrophism. In our previous work using untargeted metabolomics in Indian patients with AD, we found significant disruption in branched-chain amino acid metabolism, and *Bacopa* consumption for 12 weeks has been shown to alter metabolites of this pathway in healthy adults [33]. We intend to gain better mechanistic insights through the rigorous RCT design of this study and also correlate metabolomic changes with cognitive outcomes.

### Strengths and Limitations

The strengths of the study include a double-blind, randomized, placebo-controlled trial design, which is considered the gold standard for evaluating intervention efficacy, clearly defined relevant predictor variables and primary and secondary outcomes; a standardized and culturally validated cognitive battery tailored for the Indian population; and the use of standardized *B. monnieri* (with 50% bacosides) formulation and a matching placebo, ensuring consistency in dosage and bioactive content across participants. The second assessment at 24 weeks (12 wk after the intervention is stopped) will inform about the sustainability of cognitive changes and differences, if any, in the 2 groups. Finally, integration of metabolomics with the clinical outcomes will provide mechanistic insights on the actions of *B. monnieri* on cognition. The limitations include the exploratory nature of the study, lack of sufficient power to evaluate the efficacy, lack of biomarkers to confirm the etiological diagnosis of AD, and a relatively short follow-up period to observe meaningful change in cognitive functions.

### Future Work

Future studies could look at the efficacy and mechanisms of *B. monnieri* Linn in a larger sample through multicenter studies, using a longer duration of intervention (12‐18 mo), in a more precisely selected patient population using blood biomarkers, in genetically diverse phenotypes (eg, APOE4 positive and negative), and in the preclinical phase as a neuroprotector.

### Conclusion

To conclude, this study aims to evaluate the effectiveness of *B. monnieri* in improving cognitive performance, particularly memory, and resultant changes in blood metabolites over a 12-week intervention period in middle-aged and older Indian adults with aMCI or early AD. The results of this study have the potential to generate evidence on the role of traditional therapies as early intervention strategies for cognitive decline, particularly in conditions for which conventional treatments are unavailable.

We plan to disseminate the results of this study through scientific meetings and publication in scientific journals. The results will also be conveyed to the public in simple language during patient awareness programs after study completion. The SPIRIT 2025 reporting guidelines were used in reporting this study protocol (Checklist 1).

## Supplementary material

10.2196/82891 Multimedia Appendix 1 Supplementary tables.

10.2196/82891 Checklist 1 SPIRIT 2025 checklist.

10.2196/82891 Peer Review Report 1 Peer-review report from the Indian Council of Medical Research.

## Abbreviations

ACE-III: Addenbrooke’s Cognitive Examination III
AD: Alzheimer disease
aMCI: amnestic mild cognitive impairment
LC-MS: liquid chromatography-mass spectrometry
MCI: mild cognitive impairment
REDCap: Research Electronic Data Capture
SAE: serious adverse effect
