# Supplementary material for: Efficacy of Bacopa monnieri (Linn.) on Cognitive Function and Alterations in Blood Metabolites in Patients With Amnestic Mild Cognitive Impairment and Early Alzheimer Disease: Protocol for an Exploratory Double-Blind, Randomized, Placebo-Controlled Trial
Source: JMIR Res Protoc. 2026 Feb 25;15:e82891. doi: 10.2196/82891 (PMC12935425; doi:10.2196/82891)
Supplement: Multimedia Appendix 1 [file resprot-v15-e82891-s001.docx]

**SUPPLEMENTARY FILE**

**Table S1: Predictor variables with definitions and scales of measurement**

| **Variable** | **Definition** | **Measurement** |
| --- | --- | --- |
| Hypertension | Use of any hypertensive agents or Systolic blood pressure ≥ 140 and/or diastolic blood pressure ≥ 90 mmHg [1] | Previous medical history or  Sphygmomanometer measurement (two abnormal readings on separate occasions) |
| Type 2 Diabetes | FPG ≥ 126 mg/dl and/or 2-h plasma glucose ≥200 mg/dl and/or HbA1c≥6.5%, on 2 separate occasions– American Diabetes association (ADA) 2018 criteria [2]or using medications for diabetes | Previous medical history or  Blood Glucometer reading |
| Dyslipidemia | Presence of ‘Atherogenic dyslipidemia’ defined by a combination of High-density lipoprotein cholesterol (HDL-C) < 40 mg/dL for men or < 50 mg/dL for women and Triglycerides ≥150 mg/dL [3]; or  A high Low density lipoprotein cholesterol ≥ 100 mg/dl [4]; or  Taking lipid‐lowering medications | Blood measurements - Serum total cholesterol, triglycerides and HDL-cholesterol levels will be measured directly using an enzymatic colorimetric method with commercially available kits using an automated biochemistry analyzer. LDL-cholesterol will be calculated using the Friedewald equation except in participants with serum triglyceride ≥400 mg/dl, when a direct estimation will be made. |
| Obesity | WHO classification based on Body. Mass Index (BMI) [5].  Underweight: Below 18.5 kg/m²  Normal Weight: 18.5 to 24.9 kg/m²  Overweight: 25.0 to 29.9 kg/m²  Obesity: 30.0 kg/m² or higher | BMI will be calculated from measurements of height and weight asper formula (BMI = weight in kg/height in meters square) |
| Coronary artery disease | Yes or no | Self-reported history |
| Documented Atrial fibrillation | Yes or no | Self-reported history |
| Transient Ischemic Attack | Yes or no | Self-reported history |
| Stroke | Yes or no | Self-reported history |
| Hearing Loss | Yes or no | Self-reported history |
| Vision Loss | Yes or no | Self-reported history |
| Traumatic Brain Injury | Yes or no | Self-reported history |
| Smoking / Tobacco use | Yes or no | Self-reported history |
| Alcohol use | Yes or no  Heavy Drinking - for men, consuming more than 4 drinks on any day or more than 14 drinks per week; for women, consuming more than 3 drinks on any day or more than 7 drinks per week - National Institute on Alcohol Abuse and Alcoholism (NIAAA) [6]. | Self-reported history |
| Substance Abuse | Yes or no | Self-reported history |
| Family history of Dementia | Yes or no | Self-reported history |
| Occupation | Professional/Semi-professional/Arithmetic skill jobs/Skilled worker/Semi-skilled worker/Unskilled worker/Unemployed – B.G. Prassad socioeconomic scale updated to 2022 values [7]. | Self-reported history |
| Socioeconomic status (SES) | Upper/Upper Middle/Lower Middle/Upper Lower/Lower. SES classification derived from percapita monthly Income using B.G. Prassad socioeconomic scale updated to 2022 values[7]. | Self-reported history |

**Table S2: Changes made to protocol after CTRI submission**

| **Protocol section** | **Initial submission** | **Modification** |
| --- | --- | --- |
| Inclusion criteria | Age between 45–75 years, clinical diagnosis of amnestic MCI (NIA-AA, 2011 clinical criteria), willing to participate, give written informed consent, can read and comprehend either English or Hindi | Added ‘participant should have at-least 5 years of formal school education’ |
| Exclusion criteria | Participants with advanced malignancy, significant systemic diseases like chronic kidney disease, severe hepatic, pulmonary, cardiac disease, other neurologic diseases or drugs known to affect cognition, major surgical procedure in the last six months, TSH > 10 µU/mL at time of enrollment, major psychiatric disorder (e.g., psychosis, major depression, bipolar disorder), use of psychoactive medications, visual or auditory impairment, history of intake of Bacopa monnieri or any other investigational drug or cholinesterase inhibitors within 1 month prior to screening visit, or with alcohol abuse (National Institute on Alcohol Abuse and Alcoholism guidelines) | Elaborated on ‘other neurologic diseases or drugs known to affect cognition (like structural brain lesions, stroke, epilepsy, traumatic brain injury, Parkinson’s disease, Parkinson-plus syndromes, normal pressure hydrocephalus, autoimmune/paraneoplastic encephalitis)  Added ‘participants who have undergone a major surgical procedure in the last six months, or TSH > 10 µU/mL at time of enrollment’ |
| Allocation concealment | Sequentially numbered opaque sealed envelopes | ‘Prelabelled medicine bottles (intervention and placebo) with sequential IDs based on the randomization list’. Labelling will be done by the statistician who has generated the randomization list, and is not involved in data collection. |
| Cognitive assessments | Modified Taylor complex figure test (MTCF)  Montreal Cognitive Assessment scale (MoCA) | Test des Neuf Images du 93(TNI-93)  Addenbrooke’s cognitive examination III (ACE-III) |
| Episodic memory composite z score | Learning over trials  Delayed recall  Delayed recognition of word lists  MTCF delayed recall | Replaced ‘MTCF delayed recall’ by Immediate recall, Free recall, Cued recall and Spatial recall of TNI-93 |
| Clinically significant improvement | ≥ 2 points improvement in MoCA score | ≥ 5 points improvement in ACE-III score |
| Date of first enrollment | 06.01.2022 (Tentative) | 01.10.2023 (updated) |

**Table S3: Baseline biochemical parameters of the participants**

| **Biochemical Parameters** | **Whole Cohort (n =60)** |
| --- | --- |
| Hemoglobin (g/dl), mean (S.D.) | 13.4 (1.3) |
| Blood Urea (mg/dl), mean (S.D.) | 28.3(8.9) |
| Serum Creatinine (mg/dl), mean (S.D.) | 0.9(0.2) |
| Serum Bilirubin Total (mg/dL), mean (S.D.) | 0.7(0.3) |
| SGOT (U/L), mean (S.D.) | 22.1(5.9) |
| SGPT (U/L), mean (S.D.) | 22.2(8.7) |
| TSH^a^ (uIU/ml), mean (S.D.) | 2.9 (1.5) |
| T3^a^(uIU/ml), mean (S.D.) | 120.9(20.5) |
| T4^a^(uIU/ml), mean (S.D.) | 8.2(1.8) |
| Total cholesterol^a^ (mg/dL), mean (S.D.) | 166.3(40.5) |
| Triglycerides^a^ (mg/dL), mean (S.D.) | 146.9(74.3) |
| HDL^a^ (mg/dL), mean (S.D.) | 42.5 (11.1) |
| LDL^a^ (mg/dL), mean (S.D.) | 93.6 (35.9) |
| Vitamin B 12^a^ (pg/ml), mean (S.D.) | 332 (238.8, 587.8) |
| Folate^a^ (pg./ml), mean (S.D.) | 7.2 (4.8, 9.4) |
| FBS^a^ (mg/dL), mean (S.D.) | 113.3 (94.5, 149.9) |
| HbA1C^a^ (mmol/mol), mean (S.D.) | 6.5 (5.8, 7.4) |
| Vitamin D3^a^ (mcg), mean (S.D.) | 24.8 (16.5, 35.2) |

^a^Data missing for one participant

**Table S4: Baseline cognitive performance of the participants**

| **Variable** |  | **Whole cohort (n=60)** |
| --- | --- | --- |
| **Addenbrooke’s Cognitive Examination (ACE –III), mean (S.D.)** | |  |
|  | Attention | 16.9 (1.3) |
|  | Memory | 21.5 (3.5) |
|  | Fluency | 8.6 (1.6) |
|  | Language | 25.3 (1.0) |
|  | Visuospatial | 13.3 (2.0) |
|  | Total score | 85.4 (6.0) |
| **Trail Making Test (TMT): time taken in seconds, mean (S.D.)** | |  |
|  | TMT Part A | 85.4(49.7) |
|  | TMT Part B | 257.5 (94.6) |
|  | TMT Part [B – A] | 170.1 (80.8) |
| **Category Fluency (CF), mean (S.D.)** | |  |
|  | Animals | 11.2 (2.5) |
|  | Food | 12.0 (3.2) |
|  | Vegetables | 12.3 (2.9) |
|  | Total CF | 35.3 (6.6) |
| **Phonemic Fluency, mean (S.D.)** | |  |
|  | Ka | 6.6 (3.4) |
|  | Pa | 7.2 (3.3) |
|  | Ma | 7.0 (3.2) |
|  | Total PF | 20.8 (9.1) |
| **Verbal Learning Test (VLT), mean (S.D.)** | |  |
|  | Total Learning | 14.3 (2.5) |
|  | Learning Over Trials, | 2.1(2.7) |
|  | Delayed Recall | 2.1(1.8) |
|  | Delayed Recognition | 17.1 (2.3) |
| **Test des Neuf Images du 93 (TNI -93), mean (S.D.)** | |  |
|  | Immediate Recall | 5.6 (1.9) |
|  | Free Recall | 5.9 (1.8) |
|  | Cued Recall | 8.2 (1.4) |
|  | Spatial Recall | 6.1 (2.4) |

**Table S5: Studies on Bacopa monnieri Linn. in healthy participants & MCI or MCI – AD in the last 15 years**

| **Author, Year, Country** | **Research Design** | **Participant**  **Group** | **Mean Age** | **Property of Drug** | **Dosage** | **Duration** | **Outcomes** | **Result** |
| --- | --- | --- | --- | --- | --- | --- | --- | --- |
| McPhee et al, 2021, Australia, United Kingdom[8] | Randomized, double‑blind, placebo-controlled trial | Twenty-eight healthy adults over 55 years | 66.9±5.9 years | Bacopa Monnieri extract | Two capsules of 160 mg of Bacopa extract equivalent to 2.16 g of dried herb  Comparator – placebo  All participants completed 2-3, 60 minute sessions of online cognitive training per week | Twelve weeks | Cognitive tasks, life-satisfaction, memory complaints and mood were assessed, blood was analyzed for serum brain-derived neurotrophic factor (BDNF) before and after 12-weeks of the intervention. Diffusion tensor imaging (DTI) and neurite orientation dispersion and density imaging (NODDI) in gray (GM) and white matter (WM) were also analyzed. | The study showed mixed results. Bacopa group showed slow response times, but responses were more accurate. Exploratory neuroimaging analysis postulated an increase in network complexity through higher dendritic branching in the Bacopa group. No significant differences in biochemical biomarkers. |
| Minale et al, 2021, Thailand[9] | Randomized placebo-controlled intervention study with placebo run-in period | Forty-eight healthy volunteers | Between 55 and 80 years of age | Bacopa Monnieri extract | 300 mg of standardized Brahmi extract | Twelve weeks | Metabolites in the plasma, urine, and feces using an LC-MS--based metabolomics approach | The aminoacyl-tRNA, aromatic amino acids, and branched-chain amino acid biosynthetic pathways were mainly related to the identified metabolites in all three samples. |
| Prabhakar et al., 2020,India [10] | Randomized, double‑blind, parallel‑group, phase‑2 single‑centre clinical trial | Forty-eight patients with AD and MCI-AD | 70.2±6.7 years | Standardized mixture containing Bacosides A& B | Bacopa Monnieri was administered once daily at a  dose of 300mg for 12 months  Comparator – Donepezil 10 mg | Fifty-two weeks | The primary result was the difference in change in ADAS-Cog and PGI memory scale scores from baseline to 12 months of treatment between the two treatment groups. Patients were, however, monitored for changes in scores after 3, 6, and 9 months of treatment. Secondary outcomes were change from baseline in neuropsychological tests such as the verbal fluency-controlled oral word test and the animal names test, quality of life-Alzheimer disease, activities of daily living inventory, treatment adherence, and adverse events. | The study was terminated after 3 years & 9 months, after recruiting 34 patients because of slow recruitment & high dropout rate. Intention to treat analysis after adjusting for baseline confounders showed no difference in the rate of change ADAS – Cog score from baseline at any time, including the last follow up. There was no difference in the rate of change in PGI Memory scale (PGIMS) at 3, 6, & 9 months. In the most recent follow-up, there was a substantial difference in the change in total PGIMS score between Brahmi and Donepezil, but no difference in individual PGI memory scores. |
| Cicero et al.,  2017,  Italy[11] | Double blind, cross-over designed randomized controlled trial | 30elderly subjects (suspected dementia or MCI- AD) with  basal Mini-Mental State Examination score between 20 and 27 and self-perceived cognitive decline | 66 years | Combination of nutraceuticals based on Bacopa monnieri (dried extract), L-theanine, Crocus sativus, copper, folate and vitamins of B and D group | Bacopa monnieri 320 mg, 1 capsule, per day for 8 weeks.  Comparator - placebo | Two months | Patients were evaluated with Mini-Mental State Examination (MMSE), Perceived Stress Questionnaire (PSQ),  Index & Self-Rating Depression Scale (SRDS) at baseline and at 2 months post treatment | The active treatment arm showed considerable improvement in MMSE & PSQ Index compared to both the baseline & parallel arms. Both groups encountered a considerable  Improving the SRDS score. |
| Sadhu et al., 2014, India [12] | Randomized double-blind placebo- and active controlled clinical trial | 109 Healthy participants (Group A -–given placebo, Group B – test formulation) and 123 Patients of Senile dementia of Alzheimer’s type (SDAT) [Group C – Donepezil, Group D – Test formulation] | 60 – 75 years | Polyherbal formulation containing extract of Bacopa monnieri(whole plant), Hippophaerhamnoides, Dioscoreabulbifera | Test formulation 500 mg/day twice a daily for twelve months  Comparator – Placebo and Donepezil 10 mg twice daily | Twelve months | Cognitive, depression and functional activity scores. | After a 12-month period, administering a polyherbal formulation containing extracts of B. monnieri (whole plant) significantly improved cognitive functions in healthy elderly subjects when compared to placebo and was comparable to Donepezil in SDAT patients. |
| [Sathyanarayanan](javascript:;) et al., 2013, India [13] | Randomized, double-blind, placebo-controlled parallel design | Seventy-two healthy urban adults | 42.1±6.9 years (35-60 years) | Dried herb | Bacopa monnieri 450 mg capsule, per day for 12 weeks  Comparator - Placebo | Twelve weeks | Word list learning test (learning rate, total learning, proactive and reactive interference), inspection time, visual information processing& interference on Stroop test, Strait Trait anxiety inventory | No difference in the two groups on any cognitive measures. Trend for lower anxiety in the Bacopa group. |
| Peth-Nui et al., 2012, Thailand[14] | Randomized, double-blind, placebo-controlled parallel design | Sixty healthy elderly subjects | 62.6±6.5 years | Dried extract | Bacopa monnieri 300 or 600 mg | Twelve weeks | Working memory using word/picture presentation, simple reaction time, digit vigilance task, choice reaction time, spatial/numeric working memory; Latency and amplitude of N100 and P300 event related potentials (ERP), Acetylcholinesterase (AChE) and Monoamine oxidase activity, at baseline, every four weeks during intervention and four weeks post intervention cessation | Bacopa monnieri treated group showed improved working memory, reduced ERP latency and suppression of plasma AChE activity. |

MCI-Mild cognitive impairment, AD- Alzheimer’s disease, ADAS Cog - Alzheimer’s disease assessment scale – cognitive subscale

References:

**1.** Writing Committee Members, et al. 2025 AHA/ACC/AANP/AAPA/ABC/ACCP/ACPM/AGS/AMA/ASPC/NMA/PCNA/SGIM Guideline for the prevention, detection, evaluation and management of high blood pressure in adults: A report of the American College of Cardiology/American Heart Association Joint Committee on Clinical Practice Guidelines. Hypertension. 2025 Oct;82(10):e212-e316.doi.org/10.1161/CIR.0000000000001356.PMID:40811516.

**2.** American Diabetes Association Professional Practice Committee. 2. Diagnosis and classification of diabetes: Standards of care in diabetes-2025. Diabetes Care. 2025 Jan 1;48(1 Suppl 1):S27-S49.doi: 10.2337/dc25-S002.PMID:39651986.

**3.** Kazibwe R, Jehopio J, Schaich CL, Rikhi R, Mirzai S, Chevli PA, et al. Atherogenic dyslipidemia and incident cardiovascular events in high-risk hypertension. Prog Cardiovasc Dis. 2025 Sep-Oct;92:121-127.doi: 10.1016/j.pcad.2025.05.006. PMID:40393568.

**4.** Sawhney JP, Ramakrishnan S, Madan K, Ray S, Jayagopal PB, Prabhakaran D, et al. CSI clinical practice guidelines for dyslipidemia management: Executive summary. Indian Heart J. 2024 Mar;76 Suppl 1(Suppl 1):S6-S19.doi: 10.1016/j.ihj.2023.11.271.PMID:3805268.

**5.** World Health Organization. Body mass index. Global Health Observatory. Accessed 2025-12-14. Available from: <https://www.who.int/data/gho/data/themes/topics/topic-details/GHO/body-mass-index>.

**6.** National Institute on Alcohol Abuse and Alcoholism. Alcohol drinking patterns. Accessed 2025-08-15. Available from:

[https://www.niaaa.nih.gov/alcohols-effects-health/alcohol-drinking-patterns.](https://www.niaaa.nih.gov/alcohols-effects-health/alcohol-drinking-patterns.7)

[7](https://www.niaaa.nih.gov/alcohols-effects-health/alcohol-drinking-patterns.7). Pentapati SS, Debnath DJ. Updated BG Prasad’s classification for the year 2022. J Family Med Prim Care. 2023 Jan 1;12(1):189-190. doi: [10.4103/jfmpc.jfmpc_1478_22](https://doi.org/10.4103/jfmpc.jfmpc_1478_22).PMID:37025231.

**8.** McPhee GM, Downey LA, Wesnes KA, Stough C. The neurocognitive effects of Bacopa monnieri and cognitive training on markers of brain microstructure in healthy older adults. Front Aging Neurosci. 2021 Feb 22;13:638109. doi:10.3389/fnagi.2021.638109.PMID:33692683.

**9.** Minale G, Saesong T, Temkitthawon P, Waranuch N, Nuengchamnong N, Chootip K, et al. Characterization of metabolites in plasma, urine and feces of healthy participants after taking brahmi essence for twelve weeks using LC-ESI-QTOF-MS metabolomic approach. Molecules. 2021;26(10):2944. doi:10.3390/molecules26102944.PMID:34063409.

**10.** Prabhakar S, Vishnu VY, Modi M, Mohanty M, Sharma A, Medhi B, et al. Efficacy of Bacopa monnieri (brahmi) and donepezil in Alzheimer's disease and mild cognitive impairment: A randomized double-blind parallel phase 2b study. Ann Indian Acad Neurol. 2020 Nov-Dec;23(6):767-773. doi:10.4103/aian.AIAN_610_19.PMID:33688125.

**11.** Cicero AF, Bove M, Colletti A, Rizzo M, Fogacci F, Giovannini M, et al. Short-term impact of a combined nutraceutical on cognitive function, perceived stress and depression in young elderly with cognitive impairment: A pilot, double-blind, randomized clinical trial. J Prev Alzheimers Dis. 2017;4(1):12-15. doi:10.14283/jpad.2016.10.PMID:29188854.

**12.** Sadhu A, Upadhyay P, Agrawal A, Ilango K, Karmakar D, Singh GP, et al. Management of cognitive determinants in senile dementia of Alzheimer's type: Therapeutic potential of a novel polyherbal drug product. Clin Drug Investig. 2014 Dec;34(12):857-869. doi:10.1007/s40261-014-0235-9.PMID:25316430.

**13.** Sathyanarayanan V, Thomas T, Einöther SJ, Dobriyal R, Joshi MK, Krishnamachari S. Brahmi for the better? New findings challenging cognition and anti-anxiety effects of brahmi (Bacopa monniera) in healthy adults. Psychopharmacology (Berl). 2013 May;227(2):299-306. doi:10.1007/s00213-013-2978-z.PMID: 23354535.

**14.** Peth-Nui T, Wattanathorn J, Muchimapura S, Tong-Un T, Piyawatkul N, Rangseekajee P, et al. Effects of 12-week Bacopa monnieri consumption on attention, cognitive processing, working memory, and functions of the central nervous system in healthy elderly volunteers. Evid Based Complement Alternat Med. 2012;2012:606424. doi:10.1155/2012/606424.PMID:23320031.
