# Supplementary material for: Efficacy of Bacopa monnieri (Linn.) on Cognitive Function and Alterations in Blood Metabolites in Patients With Amnestic Mild Cognitive Impairment and Early Alzheimer Disease: Protocol for an Exploratory Double-Blind, Randomized, Placebo-Controlled Trial
Source: JMIR Res Protoc. 2026 Feb 25;15:e82891. doi: 10.2196/82891 (PMC12935425; doi:10.2196/82891)
Supplement: Peer Review Report 1 [file resprot-v15-e82891-s003.pdf]

भारतीय आयुर्विज्ञान अनुसंधान परिषद
स्वास्थ्य अनुसंधान विभाग, स्वास्थ्य एवं परिवार कल्याण मंत्रालय, भारत सरकार

**icmr**
INDIAN COUNCIL OF MEDICAL RESEARCH
Serving the nation since 1911

Indian Council of Medical Research
Department of Health Research, Ministry of Health and Family Welfare, Government of India

**संख्या:** 5/4-5/3/7/Neuro/2022-NCD-I **दिनांक:** 6/8/25

**सेवा में**

डॉ. अनु गुप्ता,
सहायक प्रोफेसर,
न्यूरोलॉजी विभाग,
एम्स, नई दिल्ली

**विषय: -"संज्ञात्मक कार्यों और अम्नेस्तिक माइल्ड कॉग्निटिव इम्पेयरमेंट तथा प्रारंभिक अल्जाइमर रोग वाले रोगियों में रक्त चयापचयों में परिवर्तन पर *Bacopa monnieri Linn.* की प्रभावकारिता: एक डबल-ब्लाइंड, रैंडमाइज्ड, प्लेसीबो-नियंत्रित परीक्षण"**

महोदया

दिनांक 14.07.2025 ईमेल का सन्दर्भ लें। उपरोक्त विषय से संबधित प्रोजेक्ट के प्रपोजल पर पि आर सी द्वारा जो कमैंट्स दिए गए थे वह संलग्न है।

भवदीय

दिनेश सोनी
06/8/2025

(दिनेश सोनी)

वरिष्ठ प्रशासनिक अधिकारी

**Proposal ID:** 2021-14050

**Title of the project:** Efficacy of Bacopa monnieri Linn. on cognitive functions & alterations in blood metabolites in patients with amnestic mild cognitive impairment and early Alzheimer Disease: A double-blind, randomized placebo-controlled trial.

**PI:** Dr Anu Gupta, AIIMS, Delhi

**Observations of PRC:**

No details on randomization. TBI-ICP/CPP- how frequently monitored? Primary and secondary outcomes not specified. Sample size not calculated, arbitrary sample no. has been selected randomly. Design is not well defined. Dose and frequency of interventional drug is not established. There is no complete information on safety and efficacy of the intervention drug drugs. Therefore there is a need for phase 2 trial not phase 3 using different doses.

**Proposal ID:**2021-14050

**Title of the project:** Efficacy of Bacopa monnieri Linn. on cognitive functions & alterations in blood metabolites in patients with amnestic mild cognitive impairment and early Alzheimer Disease: A double-blind, randomized placebo-controlled trial.

**PI:** Dr Anu Gupta, AIIMS, Delhi

**Observations of PRC:**

**COMMENT#1: No details on randomization.**

**REPLY#1:**

Stratified randomization will be done by computer generated software by statistician at CIMR, AIIMS. The randomization will be stratified based on age groups (45-60 years and 61-75 years) and randomly divided in to two groups with an allocation ratio of 1:1.

The random number sequence will be generated by an independent statistician who is not concerned with treatment in the study. Allocation concealment will be done by providing unique sequential code in opaque sealed envelopes. The code will be given on each patient's medication kits linking the kits to the randomization list. The unique sequential code will be clearly noted in patient's case report forms (CRF). The random number sequence and the randomization envelopes will be kept by the person not involved in the study. In any circumstance, the randomization list will not be shared to the Investigators and their team. The medication kits will be then presented to the clinician and the treatment will be assigned as per unique sequential code. Randomization will be done only after the participants have satisfied the eligibility criteria, and have undergone baseline evaluation. A registered Ayurvedic physician, who is a part of Investigator's team and blinded to group assignments will dispense the study medication at baseline and 6 weeks according to their unique sequential code to the patients.

(Highlighted on page no 9-10)

**COMMENT#2:** TBI-ICP/CPP- how frequently monitored?

**REPLY#2:** Traumatic brain injury or increased cranial pressure conditions are not related to the current proposal

**COMMENT#3:** Primary and secondary outcomes not specified.

**REPLY#3:**

**Primary Outcome:**

Change in the composite Z score of episodic memory (including the following scores: Learning

Over Trials, Delayed Recall, Delayed recognition of word lists, Modified Taylor Complex Figure delayed recall) in the two groups at 12 weeks

**Secondary outcomes:**

1. Identification of metabolites, pathways and networks in MCI or early AD patients after administration of Bacopa monnieri Linn at baseline and 12 weeks

2. Change in composite z score of episodic memory in the two groups at 24 weeks

3. Change in composite z score of other cognitive domains (attention, executive functions, visuospatial ability and information processing speed) at 12 weeks and 24 weeks

4. Proportion of patients with clinically significant improvement (defined as either >= 2 points improvement in MoCA, OR >=0.5 unit improvement in composite episodic memory z score, OR

a statistically significant change in the ADL score) in the two groups at the end of 12 weeks and 24weeks

5. Frequency of self-reported adverse events in the two groups during the study period

(Highlighted on page no 8-9)

**COMMENT#4: Sample size not calculated, arbitrary sample no. has been selected randomly**

**REPLY#4:**By considering a clinically relevant effect size as a S.D. change of 0.5, two-sided alpha at 5%, and 80% power, a sample size of 128(64 in each group) is calculated by statistical

software GPower (ver.3.1). With a 20% dropout rate, the final sample size reaches 160(80 in each group), which does not seem feasible for the selected patient population in a specified duration.

Therefore, a sample size of 60 (30 in each group) is determined for this exploratory study based on feasibility. Sample size is further adjusted for a dropout rate of 20%. 76(38 in each group) participants will be recruited for this study.

(Highlighted on page 9-10)

**COMMENT#5:** Design is not well defined.

**REPLY#5:** Study Design:

- Study Design: Double blind, parallel, randomized placebo-controlled trial
- No of groups: Two (Intervention and Control)
- Sample Size: 76 (38 in each group)
- Randomization: Computer generated block randomization
- Stratification: Based on age group (45-60 years and 61-75 years)
- Allocation Ratio:1:1
- Allocation Concealment: sequentially numbered opaque sealed envelope
- Study sites: Dept of Neurology, Center for Integrative Medicine & Research (CIMR), AIIMS, New Delhi.
- Study Duration: 36months

The current study will be a single center double-blind, parallel-group, randomized, placebo-controlled clinical trial with three assessment points (Baseline, 12 weeks and 24 weeks). The subjects will be recruited from the Neurology OPD, after initial screening and informed consent.

(Highlighted on page no 7)

**COMMENT#6:** Dose and frequency of interventional drug is not established.

**REPLY#6:**

We plan to use 300 mg of standardized extract of Bacopa monnieri once a day. The following studies have evaluated effect of Bacopa monnieri extracts (300mg dose) once a day on cognitive functions.

- Stough et al[1]., has evaluated effect of 300 mg of Bacopa monnieri extract daily among 46 healthy volunteers 18-60 years old, on a battery of cognitive tests after 5 weeks and after 12 weeks. The results revealed that Bacopa monnieri significantly improved speed of visual information processing measured by the IT task, learning rate and memory consolidation measured by the AVLT, and state anxiety compared to placebo, with maximal effects evident after 12 weeks.
- In a twelve weeks study by Roodenry et al[2], the effects of Bacopa capsules (300 mg for persons under 90 kg, and 450 mg for persons over 90kg, respectively)on the retention of new information in delayed recall of word pairs were also observed among 76 healthy adults aged 40 to 65years.
- Prabhakar et al[4]conducted trials evaluating effect of Bacopa monnieri (Brahmi) 300 mg OD on early AD patients for 52 weeks.

(Highlighted on page no 5)

References

1. Stough, C. et al. The chronic effects of an extract of Bacopa monniera (Brahmi) on cognitive function in healthy human subjects. Psychopharmacology (Berl). 156, 481–484 (2001).

2. Roodenrys, S. et al. Chronic effects of Brahmi (Bacopa monnieri) on human memory. Neuropsychopharmacology 27, 279–281 (2002).

3. Prabhakar, S. et al. Efficacy of bacopa monnieri (Brahmi) and Donepezil in Alzheimer's Disease and mild cognitive impairment: A randomized double-blind parallel Phase 2b study. Ann. Indian Acad. Neurol. 23, 767–773 (2020).

| S.N. | Author | Population (n) | Duration | Dosage & Frequency | Results | Safety |
|---|---|---|---|---|---|---|
| 1 | Stough et al., 2001 | Healthy volunteers (n=46) randomized to B. monniera | 12 weeks | B. monniera-capsules (150 mg) twice a day for 12 | B. monniera significantly improved speed of visual information processing measured by the IT task, learning rate and memory consolidation measured by the AVLT ($P<0.05$), and state anxiety ($P<0.001$) | Overall safe-Percentage of minor adverse effects was |

| | | | | | | |
|---|---|---|---|---|---|---|
| | | or placebo | | weeks | compared to placebo, with maximal effects evident after 12 week | similar for both treatment conditions |
| 2 | Roodenry et al., 2002 | Healthy volunteers (n=76) randomized to B. monniera or placebo | 12 weeks | 300 mg for persons under 90 kg, and 450 mg for persons over 90 kg | The results show a significant effect of the Brahmi on a test for the retention of new information. Follow-up tests showed that the rate of learning was unaffected, suggesting that Brahmi decreases the rate of forgetting of newly acquired information. Tasks assessing attention, verbal and visual short-term memory and the retrieval of pre-experimental knowledge were unaffected. | no systematic attempt was made to document any adverse reactions to the capsules |
| 3 | Prabhakar et al., 2020 | AD and MCI-AD (n=48) | 52 weeks | Bacopa monnieri (brahmi) 300 mg OD and donepezil 10 mg OD for 12 months | showed no difference in the rate of change in ADAS-Cog score from baseline at any time point, including the last follow-up. There was no difference in the rate of change in PGI Memory scale (PGIMS) at 3, 6, and 9 months. In the last follow-up, there was a significant difference in the change in total PGIMS score between brahmi and donepezil, while there was no difference in individual scores of the PGI memory scale | There was no significant difference in the number of participants with one or more adverse events |

**COMMENT#7:** There is no complete information on safety and efficacy of the intervention drug drugs. Therefore there is a need for phase 2 trial not phase 3 using different doses.

**REPLY#7:**

Following studies have reported the efficacy of Bacopa monnieri (L.)

- In a twelve weeks study, Roodenrys, S. *et al.*[1]evaluated the effects of Bacopa on the retention of new information in delayed recall of word pairs were also observed among 76 healthy adults aged 40 to 65years.
- Raghav et al[2] has reported that **Bacopa monnieri** is a relatively safe and effective drug for the treatment of age-associated memory impairment (AAMI). However they have also suggested the need for longer duration, large, double-blind studies are needed to confirm these observations.

(Highlighted on page no 4)

**References**

1. Roodenrys, S. et al. Chronic effects of Brahmi (Bacopa monnieri) on human memory.

Neuropsychopharmacology 27, 279–281 (2002).

2. Raghav, S., Singh, H., Dalal, P. K., Srivastava, J. S. & Asthana, O. P. Randomized controlled trial of standardized Bacopa monniera extract in age?associated memory impairment. Indian J. Psychiatry 48, 238–242 (2006).

| S.N. | Author | Population (n) | Duration | Dosage & Frequency | Results | Safety |
|---|---|---|---|---|---|---|
| 1 | Roodenry et al., 2002 | Healthy volunteers (n=76) randomized to B. monniera or placebo | 12 weeks | 300 mg for persons under 90 kg, and 450 mg for persons over 90 kg | The results show a significant effect of the Brahmi on a test for the retention of new information. Follow-up tests showed that the rate of learning was unaffected, suggesting that Brahmi decreases the rate of forgetting of newly acquired information. Tasks assessing attention, verbal and visual short-term memory and the retrieval of pre-experimental knowledge were unaffected. | no systematic attempt was made to document any adverse reactions to the capsules |
| 2 | Raghav et al., 2006 | Subjects above 55 years of age with complaints of memory impairment (n=40) | 12 weeks | 125 mg of SBME or placebo twice a day for a period of 12 weeks | Bacopa monniera extract (SBME) produced significant improvement on mental control, logical memory and paired associated learning | No major side-effects of the drug was reported |

**Safety of Bacopa monnieri –**

- Pravina et al[1] in a phase I study, evaluated the short-term safety and tolerability of Bacopa monniera (BacoMindTM) in healthy adult volunteers. The study used a randomized, open label, dose-escalation design. The standardized extract of Bacopa monnieri was given to each participant in a dose of 300 mg per day for 15 days and then 450 mg per day for another 15 days. The pre and post-treatment examination of clinical and hematological, biochemical, and electrocardiographic parameters of the treated volunteers did not indicate any untoward effects. There were mild adverse events related to the gastrointestinal system in the trial, which resolved spontaneously. At the dose administered for the given duration of the trial period, Bacopa monnieri extract was found to be safe in healthy adults.

(Highlighted on page no 4-5)

**References**

1. Pravina, K. et al. Safety evaluation of BacoMindTM in healthy volunteers: A phase I study. Phytomedicine 14, 301–308 (2007).

| S.N. | Author | Population (n) | Duration | Dosage & Frequency | Results | Safety |
|---|---|---|---|---|---|---|
| 1 | Pravina et al., 2007 | Healthy adults volunteers (n=23) | 30 days | 1 single capsule of BacoMindTM daily for 30 days, i.e., 300 mg for first 15 days and 450 mg for next 15 days. | Detailed examination of clinical, hematological, biochemical and electrocardiographic parameters done in pre and post treatment periods did not indicate any untoward effects in any of the treated volunteers. | Mild adverse events related to gastrointestinal system were observed in the trial, which subsided spontaneously. |

Based on the studies, there is evidence of use of Bacopa monnieri in different dosage including the intended 300 mg OD dosage in healthy adults, MCI and early AD patients. The above studies have also found Bacopa monnieri to be safe for administration. Hence we have planned this RCT using 300 mg extract of Bacopa monnieri in a OD dosage.
